# Medical Education and State License Examinations

**Published:** 1897-01

**Authors:** William Warren Potter

**Affiliations:** Buffalo, N. Y., Examiner in obstetrics, New York State Medical Examining and Licensing Board


### MEDICAL EDUCATION AND STATE LICENSE EXAMINATIONS.

Conducted by WILLIAM WARREN POTTER, M. D., Buffalo, N. Y.,
Examiner in obstetrics, New York State Medical Examining and Licensing Board.

### THE OHIO MEDICAL PRACTICE ACT.

THE Ohio State Board of Medical Registration and Examination (*Ohio Med. Jour.*) has nearly finished its labors, having registered up to the present 6,567 graduates of medicine and 588 legal practitioners, a total of 7,155 ; 136 applications have been rejected, and 100 are still pending, making a grand total of 7,491. The total number of names given in Polk's directory of physicians in Ohio is 7,560. Consequently, it is evident that the law has been very generally complied with, as very few physicians remain who have not registered.

---

### MEDICAL PROSTITUTION.

ANOTHER Cheap John medical shop (*Pacific Med. Jour.*) is being or is about to be organised in San Francisco. The medical degenerates must be accumulating in our midst. They seem to be using what little knowledge and ability they possess for the degradation and prostitution of an honorable profession. Not satisfied with offering their services to this, that and the other order or lodge, they are now preparing to organise a health insurance society, which will offer its members a year's medical treatment for $5. It is not to be presumed, however, that many members will survive a whole year's treatment from, we can hardly say a physician, perhaps doc. will answer, who values his services at considerably less than one cent and a half per visit. Our medical societies must see to it that their members take no part in such unprofessional, undignified and humiliating transactions. The professional man, in any profession, who abandons all the permanent advantages of professional standing for temporary gain, makes the blunder of his life. We commend to our societies the resolutions adopted by the medical gentlemen of Santa Clara county.

---

### REQUIREMENTS IN MISSOURI.

THE Missouri State Board of Health (*Am. Jour. Surg. and Gynec.*) has taken a stand in advance of all other states, excepting New York, in the matter of requiring every medical student to be possessed of a good education before entering any of the medical schools of the state. And the registrars are not to be judges of whether a "diploma" from a "high school," or "college," is sufficient evidence of the required education; for, hereafter, all prospective students in medical colleges will be compelled to submit to the state superintendent of schools their diplomas from other schools, to be passed upon before the applicant can take a course in medicine. Those who do not possess diplomas from high schools, or institutions whose curriculum is equivalent to the best high schools, will be barred. The request of several Kansas City medical colleges, that students be given a year after entering to complete their course in Latin, was refused by the board, and it was ruled they must be proficient in Latin before entering upon the study of medicine.

The effect will be, as the secretary, Dr. Willis P. King, of Kansas City, says:

> When a license to practise medicine is granted in this state hereafter, the recipient will be a man of education and refinement, and no stable hands or car drivers will write "M. D." after their names. These fellows who get a diploma from a school taught by Bill Jones and his wife, down in Bear Creek, cannot become physicians, unless the superintendent of schools decides they have acquired more than the rudiments of education. We may freeze out some of these inferior colleges, or reduce the number of graduates, but it will be a good thing, and the death-rate will be lower.

And all progressive, fair-minded doctors will be thankful that it is so! Now, let some of the neighboring states follow our example. Unless they do so, they will soon be overrun with the illiterates.

### AN INSPECTOR OF MEDICAL SCHOOLS.

A PHYSICIAN of Baltimore (*Md. Med. Jour.*), who has the good of medical education at heart, has made the very sensible and practical suggestion that there should be an inspector of medical schools to see that the required standard was rigidly adhered to, and to compare the facilities offered with the enticing statements made in the catalogues and annual circulars.

---

### RAILWAY SURGEONS ARE EXEMPT.

WE QUOTE a few legal points (*International Jour. Surg.*) from an article by Dr. W. D. Hawaker, of Meadville, Pa., in the *New York Medical Journal*, July 18, 1896 :

Pennsylvania has a liberal yet very efficient law governing the practice of medicine in that state—a law which many of our states would do well to examine and adopt. The framers of the Pennsylvania law foresaw the necessity of reciprocity and have provided for it.

"The law of 1893 has been in force since June, 1894, and has for its main object the examination of all who wish to begin the practice of medicine and surgery in Pennsylvania.

"This law establishes a medical council, composed of the lieutenant-governor, the attorney-general, the secretary of internal affairs, the superintendent of public instruction, the president of the state board of health, and the presidents of the *three* state boards of medical examiners.

"The council issues all licenses to practise, and all candidates for examination and license write to the secretary of the council at Harrisburg, presenting their credentials, and receive permission from him to appear before one of the state boards of examiners.

"Physicians licensed by boards of other states are licensed in Pennsylvania, on filing their licenses, duly certified, together with *proper affidavits showing the standards of the other States to be equal* to that of Pennsylvania.

"Medical officers of the army and navy, marine hospital, *and railroad relief departments*, internes of hospitals, dentists, druggists, and instrument-makers are exempted.

"Physicians of other states can come into the state in consultation with Pennsylvania physicians, and physicians just across the line in other states whose practice extends over into Pennsylvania, can attend their patients, but must not have an office or place to meet patients in this state.

"Practitioners duly registered before March 1, 1894, shall not be interfered with; and one such registry shall be sufficient warrant to practise medicine and surgery in any county in this commonwealth."

---

### AN ITINERANT PHYSICIAN NOT ENTITLED TO PRACTISE.

THE Supreme Court of Rhode Island, February 11, 1896, (*St. Louis Med. and Surg. Jour.*,) affirmed the decision in the case of Evans vs. State Board of Health, that here was a party who was to be regarded as an itinerant doctor within the meaning of the statute relating to the practice of medicine in that state, which provides that "nothing in this chapter shall be so construed as to authorise any itinerant doctor to register or to practise medicine in any part of this state." He was a domiciled resident of Boston, Mass., and a practising physician, making a specialty of the treatment of catarrh. His main or regular office was in Boston, and for years past, except when absent from the country or prevented by illness, he had visited Providence in the practice of his specialty on stated days each month. He had no office in Providence, except the rooms he had taken in the hotels at which he stopped. He notified his patients of his visits by advertisements in a Providence newspaper, and had met them in his rooms at the hotels at the times mentioned in the advertisements. He had also, during this ten years, for greater or less periods, been in the habit of visiting, in the practice of his specialty, Worcester, Springfield, New Bedford and Lowell, Mass., in the same manner as he had visited Providence. On these facts the court holds that the decision of the State Board of Health, as above, was correct.

---

### ENTRANCE EXAMINATIONS.

WITHIN a few weeks the medical colleges of our land (*Medical Fortnightly*) will throw open their doors for the reception of students, to start again "the grind" which leadeth to the formation of doctors of medicine. Already the registration of students is going on, and the first year man, yet to have his eye-teeth cut, swells with pardonable pride when his inside pocket contains the matriculation ticket which says that he is enrolled as a student in his chosen college, subject, however, in many cases, to his passing the required entrance examination, which takes place on his arrival at the college. This proviso, he will soon learn, is in the great majority of institutions of no consequence and merely a bluff to make him think requirements are real instead of apparent.

To a certain extent the movement inaugurated in St. Louis recently by the State Board of Health, to have united action of the various medical colleges of the city, as regards entrance examinations, will be lived up to and will confer benefit upon the profession of medicine. But knowing this fact, there never was a lock devised but that there was a means devised for cracking it; there never was a law inaugurated but that some schemer devised a plan to defeat its purposes; and so when it comes to entrance examinations, we know that while the purpose and intent of the law is good, it will here in St. Louis be stretched and distorted so that its own mother would hardly recognise it if she should meet it on the street.

So long as the spirit of commercialism and competition, which, alas! pervade the "objects and purposes" of such an organisation as a medical college, there will be a letting down of the bars, where in the best interests of the college (?) it is expedient not to draw too fine the line of demarcation between the fit and unfit for the noble, high-minded profession of medicine. We are reminded that competition is the life of trade, and a long list of matriculants and graduates in a medical college annual announcement is supposed to say to its rivals, "trade is booming in our neighborhood." To keep in the swim, the rivals must publish a list of real or straw men of equal or greater length, and the merry war is on. Now, to get matriculants there must be some inducement to draw the students; either reduced rates or easy examinations must be the attraction—probably both. These matriculants, armed with their bargain-day sale of matriculation tickets, are then gathered beneath the sheltering roof of their to-be Alma Mater, there they must present credentials for the approval of the State Board of Health, and then we see the curtain rung up on the most amusing act in the farce comedy of examinations. Think of such questions as the following being propounded as fit to determine qualifications for entrance into a medical college—namely: How long is a piece of string? What is the difference between fizz and bumfuzzle? When does a calf become a cow? If a dog and a half cost a dollar and a half, what will two dogs cost? Where was Moses when the light went out? Some will ask whether the above questions were really asked; we know to our personal knowledge that an oral examination was commenced with the first question and ended with the last. In Milwaukee they have a school in which you can graduate by answering the above questions, providing you pay $35. At Chicago's famous sun-down colleges, one of which is now in trouble with the staid and reliable Harvard University, all you have to do is to certify before a notary that you are competent to practise medicine, and you get your diploma on payment of a nominal fee. No questions asked. Now Missouri has started the ball rolling in the right direction by requiring entrance examinations, but if she wants to keep her name and fame free from tarnish she will have to watch the trap for the unfit, now that she has set it, to see that its springs work and that some fellow don't fool with the machine. We hope to see the determination of the necessary qualifications of individuals to study medicine, and to practise it, determined by a board of examiners. The present lock is a good one, but it is so easily cracked by those who know how.

---

### THE MARYLAND EXAMINING BOARD.

In this issue (*Maryland Med. Jour.*) are presented the questions with the results of examinations of the State Board of Medical Examiners of Maryland, together with a tabulated report of the work of that board since its formation in 1892.

Many persons have taken occasion to cast slurs on examining boards, saying that the men on them not being connected with medical schools have no experience in such work and ask absurd questions which they themselves would not be able to answer. Others maintain that such tests are not fair.

It must be, however, admitted that state examinations are, on the whole, a great benefit to the profession and to the public. There is attempted an equalisation of all graduates and a general standard of all physicians in one state is held. If states could agree on a national board by which any physician could practise anywhere in the United States it would be a little more fair.

In looking over the reports of any board, it is seen that graduates from some schools always stand high, with some exceptions, while other schools show a majority of rejections. The natural conclusion is that all medical schools do not maintain the same standard.

If the state board could have additional powers whereby they could demand that schools keep up to a fixed standard and give all the facilities set forth in their catalogues and prospectuses, the number of failures at the state examination might not be so large.

Dr. Chas. C. Wadsworth, secretary of the California State Board of Examiners, has removed his office and residence to 1104 Van Ness avenue, between Geary and Post streets, San Francisco.

---

NEW MEMBERS OF THE MEDICAL EXAMINING BOARD OF VIRGINIA.

To fill the vacancies caused by the deaths (*Va. Med. Semi-Monthly*), since the session of the Medical Society of Virginia in September last, of Dr. J. Edgar Chancellor, of the seventh congressional district, and of Dr. H. M. Patterson, of the tenth congressional district, the Executive Committee of the State Society has nominated for commission by the Governor of Virginia, Dr. E. M. Magruder, of Charlottesville, and Dr. Charles W. Rodgers, of Fishersville. These gentlemen are expected to fill the unexpired term until November 1, 1898. Dr. Magruder was instructor in physical diagnosis in the University of Virginia, and his abilities as a practitioner are well known to the profession. Dr. Rodgers passed the Medical Examining Board of Virginia during 1885—the first year of the going into effect of the law establishing the board.

